# Calcineurin-Inhibitor-Induced Hypomagnesemia in Kidney Transplant Patients: A Monocentric Comparative Study between Sucrosomial Magnesium and Magnesium Pidolate Supplementation

**DOI:** 10.3390/jcm12030752

**Published:** 2023-01-17

**Authors:** Lucia Federica Stefanelli, Marianna Alessi, Giovanni Bertoldi, Valentina Rossato, Valentina Di Vico, Federico Nalesso, Lorenzo A. Calò

**Affiliations:** Nephrology, Dialysis and Transplantation Unit, Department of Medicine, University of Padova, 35128 Padova, Italy

**Keywords:** kidney transplant, calcineurin-induced hypomagnesemia, sucrosomial magnesium

## Abstract

Magnesium (Mg) contributes to DNA stability, protein synthesis and cardiac excitability, while Mg deficiency leads to increased cardiovascular mortality, diabetes, hyperparathyroidism and risk of fractures. In kidney transplant patients, calcineurin inhibitors (CNIs) downregulating Mg channel TRPM6 in the distal collecting tubule induce early hypomagnesemia (HypoMg), which is associated with a faster decline in allograft function. A new formulation, sucrosomial Mg (SucrMg), for oral supplements encapsulates Mg oxide in a phospholipid membrane covered by a sucrester matrix, enhancing gastric and intestinal Mg absorption. This study has evaluated Mg bioavailability, effectiveness and tolerance of SucrMg compared to the conventional preparation of Mg pidolate (PidMg). The association of blood Mg with risk of post-transplant dysglycemia and hyperparathyroidism has also been investigated. Forty hypomagnesemic adult single, double or combined kidney–pancreas or kidney–liver transplant recipients within 2 years from transplantation were recruited. In total, 16 patients received PidMg and 27 received SucrMg. Blood Mg was measured at baseline (T0), after 15 days (T1) and after 6 months (T2) of treatment. PTH, fasting glucose and calcium were measured at baseline and after 6 months of treatment. The tolerance was evaluated at the ambulatory visits. SucrMg compared to PidMg was more efficient at increasing Mg bioavailability at T1: *p* < 0.0001 vs. *p* = 0.72 ns, respectively, with a ∆% increase of 12.4% vs. 5.4%, *p* = 0.04. Both preparations increased blood Mg at T2, *p* < 0.0001 and *p* = 0.002, respectively. SucrMg was better tolerated. No difference was observed for fasting plasma glucose, PTH and calcium. On one hand, our study is the first among transplant patients to evaluate the efficacy of SucrMg in the correction of HypoMg, which might justify the limited number of patients enrolled and the short observation time; on the other hand, our results could serve as a useful working hypothesis for further studies with a larger number of transplant patients and an extended study duration to confirm the benefits observed with SucrMg.

## 1. Introduction

Magnesium (Mg) is an essential cation and a key regulator of human health. It is in fact a cofactor for adenosine triphosphatases, and it is critical in energy-requiring metabolic processes, as well as protein synthesis and anaerobic phosphorylation. In addition, Mg plays a critical role in maintaining normal cardiac excitability, blood pressure, bone integrity, glucose and insulin metabolism [1]. In this regard, Mg deficiency has been associated with cerebrovascular events, hypertension, cardiovascular disease, type 2 diabetes and osteoporosis [2], and hypomagnesaemia (HypoMg) is associated with negative outcomes and mortality [1].

HypoMg is a common condition occurring in up to 12% of hospitalized patients, and can be the result of gastrointestinal losses, genetic diseases, volume expansion, diuretics use, epidermal growth factor receptor inhibitors, proton pump inhibitors and calcineurin inhibitors (CNIs) [3].

HypoMg is very common in the first months after kidney transplantation [4]. The high prevalence of HypoMg in the early post-transplant period is mainly attributable to CNIs and, in particular, tacrolimus. The mechanism through which HypoMg develops mainly includes renal Mg wasting, essentially via CNI-induced downregulation of the Mg transporter Transient Receptor Potential Melastatin 6 (TRPM6) in the distal collecting tubule [5]. Several studies have demonstrated an association between lower serum Mg and a faster decline in kidney function, and lower serum Mg might also be involved in mechanisms leading to delayed graft function [6].

There is a consensus on the need of Mg supplementation in HypoMg induced by CNIs. Of potential interest to our study, Mg supplementation in rats exposed to a low-salt diet and cyclosporine not only corrected HypoMg, but also prevented the expected development of renal histological damage with interstitial fibrosis and concomitant decline in kidney function [7].

HypoMg in transplant patients should be corrected with supplements. Conventional oral Mg supplements exhibit poor absorption and bioavailability, likely due to the interference of dietary compounds or digestive factors. On these bases, a sucrosomial Mg formulation (Ultramag^®^) in a sustained release matrix has been developed in order to enhance Mg bioavailability [8]. This new formulation is an innovative preparation of Mg oxide, covered by phospholipids plus a sucrester matrix, and can be used as an alternative to standard Mg salts to improve Mg supplementation efficacy. This encapsulation of Mg ions within a sucrosomial membrane allows the passage of the ions through the gastric and intestinal environment to reach the blood stream without any interaction with the intestinal mucosa [9].

No studies that evaluate Mg bioavailability, effectiveness and tolerance of Ultramag^®^ in transplant recipients are available. Our Transplant Center monocentric study examined Mg bioavailability, effectiveness and tolerance of the new formulation Ultramag^®^ in transplant patients compared to the conventional preparation of Mg pidolate (Mag2^®^) in a time period of 6 months.

The presence of an association between blood Mg level and risk of post-transplant abnormal glycemia and hyperparathyroidism was also investigated.

## 2. Materials and Methods

### 2.1. Study Population

Forty transplant recipients who received a single, double or combined kidney–pancreas or kidney–liver transplants between 2019 and 2021 at the Kidney–Pancreas Transplant Unit of Padova University Hospital were enrolled. The patients were randomized to receive either Ultramag^®^ or Mag2^®^.

All the study participants were followed at the Kidney–Pancreas Transplant Ambulatory Unit of Padova University Hospital. Treatment was started with very low levels of serum Mg (HypoMg is defined below 0.7 mmol/L or 1.7 mg/dL), developed within 6 months/1 year from transplantation. Patients’ tacrolimus trough levels were the same for both groups (between 6.5 and 8 μg/L). In total, 27 out of 40 patients were supplemented with 1 sachet of Ultramag^®^ (375 mg of Mg element)/day, while 16 were treated with 3 vials of Mag2^®^ (370 mg of Mg element/day) (Table 1). Three patients of the Mag2^®^ arm dropped out due to side effects after 1 month (diarrhea). Adult subjects (>18 years) of either gender were eligible. Other inclusion criteria were that patients must have undergone a single, double or combined kidney–pancreas or kidney–liver transplant between 2019 and 2021, and maintenance immunosuppressive therapy that consisted of CNI, mycophenolate/mTOR inhibitor and steroid, or CNI plus steroid-only treatment.

Patients were excluded if they had a history of intestinal resection, inflammatory disease of the gastrointestinal tract, malabsorption, presence of other drugs potentially affecting Mg reabsorption, such as diuretics, or genetic and familiar HypoMg.

### 2.2. Laboratory Evaluation

Venous blood samples were taken for baseline Mg level, fasting plasma glucose, parathyroid hormone (PTH) and calcium level (T0). Blood samples were also collected at 15 days after Mg supplementation (T1) and at 6 months (T2). Blood levels of PTH, fasting glucose and calcium were evaluated after 6 months of Mg supplementation.

Each patient was examined at the 15th day of Mg supplementation during the outpatient ambulatory visit, and every 2 months thereafter, in order to evaluate possible side effects of the treatments. Blood samples were analyzed at the Central Laboratory of the Padova University Hospital, Italy, using Rosch Cubas 8000 for electrolyte evaluation and LiasonXL machine for PTH. Blood Mg, Ca and fasting plasma glucose are expressed in mmol/L, while PTH is expressed in ng/L.

### 2.3. Statistical Analysis

Statistical analysis was performed on a Macintosh iMac computer using GraphPad Prism 9.0 software (GraphPad Software Inc., La Jolla, CA, USA). Data are expressed as mean ± standard deviation (SD). The Shapiro–Wilk test was used to check the normal distribution. Analysis of variance (ANOVA) was used to compare the quantitative variables between groups, and Student’s *t*-test for paired and impaired data was also used. Values of 5% or less (*p* < 0.05) were considered significant.

## 3. Results

**Figure 1:**
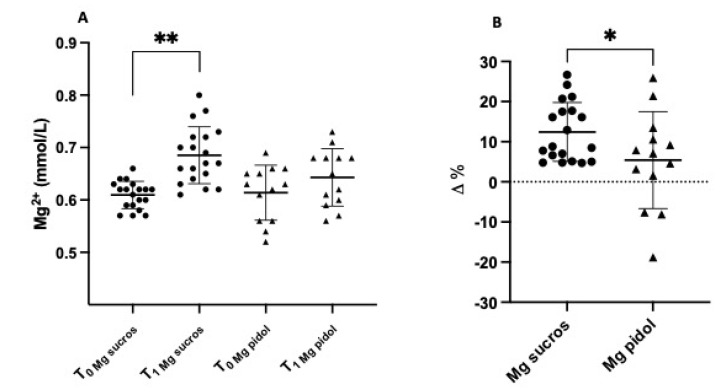
(**A**) Mg blood level in kidney transplant patients at baseline (T0) and after 15 days of treatment (T1) with Ultramag^®^ vs. Mag2^®^ and (**B**) ∆% increase in blood Mg after 15 days of treatment with Ultramag^®^ vs. Mag2^®^. ** *p* < 0.0001; * *p* = 0.04.

Treatment with Ultramag^®^ increased Mg bioavailability very early after the start of Mg supplementation. Mg level significantly rose, in fact, after 15 days of treatment from the baseline (T0) with Ultramag^®^ vs. Mag2^®^: 0.60 ± 0.02 mmol/L T0 to 0.69 ± 0.05 mmoL/L T1, *p* < 0.0001, vs. 0.61 ± 0.05 mmol/L T0 to 0.64 ± 0.05 mmol/L T1, *p* = 0.72 (ns), respectively (Figure 1A), which, in terms of ∆% Mg increase, was 12.4% for Ultramag^®^ vs. 5.4% for Mag2^®^, *p* = 0.04 (Figure 1B).

**Figure 2:**
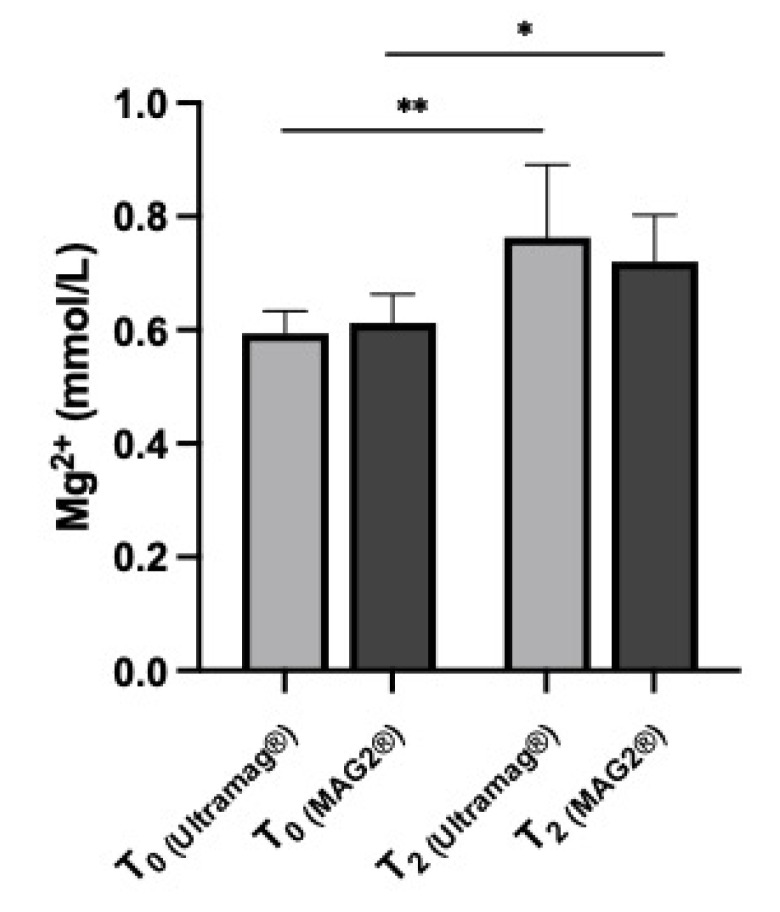
Mg blood level in kidney transplant patients at 6 months (T2) of treatment with Ultramag^®^ vs. Mag2^®^ from baseline (T0). ** *p* < 0.001; * *p* < 0.002.

Both Ultramag^®^ and Mag2^®^ provided a significant increase in Mg level at 6 months from baseline: 0.60 ± 0.02 mmol/L T0 to 0.76 ± 0.13 mmol/L T2, *p* < 0.001, for Ultramag^®^, and 0.61 ± 0.05 mmol/L T0 to 0.72 ± 0.08 mmol/L, *p* = 0.002, for Mag2^®^ (Figure 2).

The analysis of possible side effects with Ultramag^®^ and Mag2^®^ showed that one patient in the Ultramag^®^ group developed mild gastrointestinal side effects (modest nausea), whereas five patients treated with Mag2^®^ had diarrhea (three of them dropped out due to intense diarrhea) and one patient experienced nausea (Table 1).

## 4. Discussion

HypoMg (blood Mg < 0.70 mmol/L) is a common entity, particularly among hospitalized patients, with an incidence up to 60–65% in intensive care patients [10]. It can be associated with weakness, ataxia, cramps, tetany, seizures and arrythmias/electrocardiographic changes, and can be attributed to chronic disease, alcohol use, gastrointestinal and renal losses and drugs [11].

In this context, CNIs (e.g., cyclosporine and tacrolimus), the most commonly used immunosuppressant drugs, very often cause severe HypoMg early after kidney transplantation. CNIs cause renal Mg loss, likely due to the CNI-induced reduction in TRPM6 expression [5]. Recent studies have reported that the majority of kidney transplant recipients develop HypoMg within the first weeks and months after transplantation [12]. Some studies have suggested a nadir of serum Mg concentration around the second month; HypoMg persists, however, in more than 20% of kidney transplant recipients many years after transplantation. Moreover, an association between low serum Mg and a faster decline in kidney function has been reported in cohorts of kidney transplant recipients, and HypoMg might also be involved in mechanisms leading to delayed graft function [13].

Mg supplementation is a safe strategy to help prevent these consequences. Conventional oral Mg supplementation with different Mg preparations has, however, a very poor intestinal adsorption, which in turn results in modest bioavailability and limits its efficacy, in addition to having negative gastrointestinal side effect profiles [14]. A new sucrosomial Mg formulation (Ultramag^®^) has been developed with an innovative technology able to promote intestinal adsorption of Mg ions without any interaction with the intestinal mucosa, therefore increasing its bioavailability with a reduction in side effects [9].

Our monocentric study has evaluated, for the first time in transplant recipients, the effect of Mg supplementations with SucrMg (Ultramag^®^) compared with one of the conventional Mg preparations, PidMg (Mag2^®^), in order to investigate the effect of Ultramag^®^ on Mg bioavailability, efficacy and tolerability.

In patients treated with Ultramag^®^, serum Mg level compared to Mag2^®^ significantly increased after 15 days of treatment. These data are in line with the double-blind crossover study on a healthy population conducted by Brilli et al., which showed the efficacy of sucrosomial technology of Ultramag^®^ to increase Mg absorption and bioavailability [8]. However, both Ultramag^®^ and Mag2^®^ increased Mg concentration at 6 months from baseline, although Mg level with Ultramag^®^ was higher compared to that with Mag2^®^.

We also checked in the medium/long-term tolerance of patients to treatment. At the outpatient visits, the patients were accurately interviewed in order to evaluate gastrointestinal or other side effects. The results show the higher efficacy of Ultramag^®^ compared to Mag2^®^ in terms of not only bioavailability and effectiveness, but also tolerance. In fact, only 1 patient out of 27 in the Ultramag^®^ group reported adverse gastrointestinal events (modest nausea), while 6 patients out of 16 in the Mag2^®^ group developed side effects related to the treatment: 5 with diarrhea (3 patients dropped out) and 1 with modest nausea.

In the general population, low blood Mg level is associated with the development of type 2 diabetes and osteoporosis with an increase in PTH. Mg supplements increase insulin sensitivity and improve glycemic control in people with insulin resistance and diabetes [15]. Moreover, previous studies have demonstrated an association between Mg supplementation and improved blood calcium levels, alongside a reduction in PTH and an enhanced bone density status.

In kidney transplant patients, HypoMg is common, and indicates diabetes. In this regard, Van Laecke and coworkers have demonstrated that Mg supplements modestly improved fasting glycemia compared to the control group [16]. Regarding calcium–phosphorus metabolism, even after kidney transplantation, recipients can continue to exhibit elevated PTH levels; Mg supplementation also ameliorates hyperparatiroidism in kidney transplant patients [17].

In our study, neither Ultramag^®^ or Mag2^®^ showed any difference regarding glycemic control and calcium–phosphorus metabolism. In fact, PTH dropped with both Mg preparations independently of the entity of Mg correction. This could depend on the renal recovery after kidney transplantation, which improved bone and mineral abnormalities, and secondary hyperparatiroidism present during CKD. Serum calcium level was unchanged from baseline with both Ultramag^®^ and Mag2^®^. None of the patients of our study with low serum Mg concentration developed any initial hypocalcemia. However the unchanged blood calcium could be the result of the patients’ treatment with vitamin D analogs before and during transplantation.

Although pretransplant hypomagnesemia was associated with post-transplant dysglycemia, in our study, glycemic metabolism was unchanged with both Ultramag^®^ and Mag2^®^ [18]. However, the screening of new-onset diabetes after transplantation should consider the possible consequence of the induction of immunosuppressive therapy with a high dose of corticosteroids, which might promote glucose intolerance via increasing insulin resistance and reducing insulin sensitivity.

## 5. Conclusions

The results of this study, although obtained from a small cohort of transplant patients, show that Ultramag^®^ is more efficient and better tolerated compared to Mag2^®^. Ultramag was, in fact, significantly more efficient at increasing Mg bioavailability in the short term (15 days), while in the longer period (6 months), both types of Mg supplementation increased Mg levels compared to baseline, with only slightly increased Mg blood levels by Ultramag compared to Mag2. The benefits in regards to the side effect profile were instead strikingly shown by Ultramag, which was definitively better tolerated throughout the study compared to Mag2 (five patients had diarrhea, three of which dropped out due to intense diarrhea, and one experienced nausea in the Mag2^®^ group, whereas there as only one patient with modest nausea in the Ultramag^®^ group). Given the high prevalence of this electrolyte disorder in renal transplant recipients with contemporary immunosuppressive treatment, the importance of an optimal supplementation of Mg is essential. The small number of patients enrolled and the short observation time could clearly be a limitation of our study; however, although with these limitations, our results, obtained for the first time in transplant recipients, add information on the efficacy and safety of sucrosomial Mg supplementation in this type of patient with HypoMg, which was not previously available. The fact that our study is the first among transplant patients to evaluate the impact of Ultramag^®^ for the correction of HypoMg might justify the limited number of patients enrolled and the short observation time; on the other hand, our results could serve as a useful working hypothesis for further studies with a larger number of transplant patients and an extended study duration to clearly confirm the higher efficacy and tolerability of Ultramag^®^, in addition to showing, in these patients, the mediated improvement in other effects linked with pretransplant HypoMg, such as post-transplant dysglycemia, PTH levels and possible calcium–phosphorus abnormalities.

## Figures and Tables

**Table 1:** Study population.

	Ultramag^®^	Mag2^®^
Patients	27	16
Drop out	0	3
Age range (Years)	47 (22–72)	52 (35–82)
Female	9 (33.3%)	5 (38.4%)
Male	18 (66.6%)	8 (61.6%)
Patients in triple immunosuppressive therapy	26 (96%)	12 (92%)
Patients in double immunosuppressive therapy	1 (4%)	1 (8%)
Dose of Mg	375 mg (1 sachet)	370 mg (3 vials)
Baseline Mg levels	0.60 ± 0.02 mmoL/L (1.46 ± 0.05 mg/dl)	0.61 ± 0.05 mmol/L (1.48 ± 0.12 mg/dL)
Side effects	1 patient (nausea)	6 patients (3 of them dropped out for diarrhea) and 1 had nausea

## Data Availability

Not applicable.
